# Next-generation in situ hybridization approaches to define and quantify HIV and SIV reservoirs in tissue microenvironments

**DOI:** 10.1186/s12977-017-0387-9

**Published:** 2018-01-09

**Authors:** Claire Deleage, Chi N. Chan, Kathleen Busman-Sahay, Jacob D. Estes

**Affiliations:** 1 AIDS and Cancer Virus Program, Frederick National Laboratory for Cancer Research, Leidos Biomedical Research, Inc., Frederick, MD 21702 USA; 2 Present Address: Vaccine and Gene Therapy Institute, Oregon National Primate Research Center, Oregon Health & Science University, Beaverton, OR 97006 USA

## Abstract

The development of increasingly safe and effective antiretroviral treatments for human immunodeficiency virus (HIV) over the past several decades has led to vastly improved patient survival when treatment is available and affordable, an outcome that relies on uninterrupted adherence to combination antiretroviral therapy for life. Looking to the future, the discovery of an elusive ‘cure’ for HIV will necessitate highly sensitive methods for detecting, understanding, and eliminating viral reservoirs. Next-generation, in situ hybridization (ISH) approaches offer unique and complementary insights into viral reservoirs within their native tissue environments with a high degree of specificity and sensitivity. In this review, we will discuss how modern ISH techniques can be used, either alone or in conjunction with phenotypic characterization, to probe viral reservoir establishment and maintenance. In addition to focusing on how these techniques have already furthered our understanding of HIV reservoirs, we discuss potential avenues for how high-throughput, next-generation ISH may be applied. Finally, we will review how ISH could allow deeper phenotypic and contextual insights into HIV reservoir biology that should prove instrumental in moving the field closer to viral reservoir elimination needed for an ‘HIV cure’ to be realized.

## Background

Despite the robust efficacy of combination antiretroviral therapy (cART), current treatments do not provide a cure for infection with HIV, or the closely related simian immunodeficiency virus (SIV), as treatment interruption inevitably leads to uncontrolled rapid viral rebound in the vast majority of individuals and animals [1]. This predictable recrudescence occurs due to the presence of viral reservoirs, cellular or anatomical sites within the host in which infectious virus can persist in the face of antiviral immune responses and highly effective antiretroviral (ARV) treatment. Identifying and, ultimately, understanding mechanisms and principal tissue compartments for viral reservoir persistence within an infected host will be essential for the development of effective cure strategies for HIV.

A variety of quantitative approaches have been used historically to increase our understanding of viral reservoirs, each providing unique insights, but, unsurprisingly, constrained by both technical limitations and accessibility of relevant samples. For instance, much of what we know about the latent CD4^+^ T cell reservoir (i.e. frequency, slow decay rates, viral integration sites, mechanisms of persistence, etc.) has come, in large part, from analyzing peripheral blood mononuclear cells (PBMCs) isolated from HIV-infected patients on cART [2–6]. Given the ease of accessing peripheral blood, quantification of the latently infected resting CD4^+^ T cell reservoir within the PBMC compartment is often used to determine the effectiveness of various cure strategies, providing a cost-effective and minimally invasive means for monitoring viral persistence in these treatment settings. However, our increased understanding of the important role that lymphoid and select non-lymphoid tissues play in HIV infection and persistence, and the limited cellular populations that circulate through peripheral blood, necessitate more detailed and comprehensive analyses of viral reservoirs in tissue compartments and how intervention cure strategies impact these tissue reservoir populations.

While the quantification and phenotypic characterization of HIV reservoirs during the course of viral reduction strategies continues to be performed on cell suspensions or purified cellular populations using a variety of sensitive methods [7–10], these approaches lose important spatial and contextual information about viral reservoirs within their native tissue microenvironments. Since the predominant cell types infected by HIV/SIV are key components of the immune system (i.e. CD4^+^ T cells and macrophages), reservoir establishment, viral persistence, host immune responses to infection, and disease pathology all take place within tissues, which have a distinct cellular composition and function from the peripheral blood. In addition, some resident tissue cells (i.e. follicular dendritic cells and certain macrophage populations) are notoriously challenging to isolate and study ex vivo and do not circulate through the peripheral blood [11, 12]. Thus, in situ-based approaches provide important and novel insights regarding the localization and phenotypic features of reservoir cells within their resident native tissue microenvironments, which is simply not feasible by other approaches.

Traditionally, the study of HIV/SIV infection and persistence by ISH approaches was limited to only a handful of expert groups due, in part, to technical challenges with performing these kinds of studies. Visualization of viral RNA (vRNA) in formalin-fixed, paraffin embedded (FFPE) tissues was originally performed by radiolabeled ISH (R-ISH) [13, 14]. This technique is sensitive, and able to detect individual productively-infected cells as well as the abundant virions trapped on the follicular dendritic cell (FDC) network in B cell follicles (BCFs) within lymphoid tissues [15, 16]; however, R-ISH is a highly labor intensive and low-throughput technique. This approach requires the use of radioactive material and variable, but long, incubation times (typically 1–4 weeks) before generating autoradiographs with developed silver grains localized over the cells containing vRNA on the tissue sections [13, 14]. Due to these issues, coupled with a relatively low signal-to-noise ratio the longer the samples are exposed (high background due to off-target binding), subsequent ISH approaches have sought to overcome these limitations inherent with R-ISH. While chromogenic ISH (C-ISH) and fluorescent ISH (F-ISH) have eliminated the use of radioactive material and can typically be performed in around 3–4 days, these approaches have much less sensitivity than R-ISH [17, 18].

The specific and sensitive detection of low copies of vRNA, and viral DNA (vDNA), in tissue sections necessitates the generation of a reliable ISH approach for the study of HIV/SIV reservoirs and viral persistence in vivo. While additional ISH approaches have been developed in an attempt to detect low abundance vRNA and vDNA targets (i.e. in situ PCR [19, 20], tyramide amplification [21], and rolling circle amplification [22]), the usefulness of these approaches in reservoir studies has been hindered, in part, by assay complexity, low reproducibility, insufficiently high false detection rate, and/or unsuitably high background due to off-target probe binding. The recent development of a novel, highly specific, sensitive, rapid, and facile next-generation ISH approach (termed RNAscope), with sensitivity approaching single-RNA molecule visualization in individual cells [23], has provided significantly improved capabilities for imaging, phenotyping, and quantifying HIV/SIV reservoirs (and individual virus particles) in situ, leading to new and important insights into reservoir biology and viral persistence [16].

In this review, we will discuss how these next-generation, high-throughput in situ hybridization approaches can be used to provide a more comprehensive view of viral reservoirs moving forward. We will describe how these assays allow for the detection of low copy vRNA and vDNA signals in situ, enabling the visualization of rare viral reservoirs in their native tissue microenvironments. These approaches, while still new and evolving, have provided important insights in understanding reservoir establishment, persistence during cART, and should provide powerful tools needed for the assessment of the full impact of potential ‘cure’ strategies moving forward.

## In situ hybridization to detect HIV/SIV vRNA and vDNA

### RNAscope and DNAscope ISH

A new next-generation, in situ hybridization approach (RNAscope) was described in 2012 for the detection of host messenger RNA (mRNA) in FFPE cells and tissues, with a sensitivity approaching single-RNA molecule visualization in individual cells [23]. The remarkable specificity of this approach is achieved through the unique requirement for two ‘double-Z’ target probes to bind contiguously to their respective complementary RNA sequences in order for a signal preamplifier to subsequently bind, initiating a signal amplification cascade via sequential hybridization steps similar to branched-chain DNA (bDNA) [16, 23]. This ‘double-Z’ probe binding prerequisite ensures extremely low-to-no visible background due to the exceedingly low probability of two probe pairs each nonspecifically hybridizing next to each other, an essential requirement for all successive hybridization steps [16, 23]. The exquisite sensitivity of the RNAscope platform, with the ability to visually resolve single RNA molecules in tissue sections, comes from the combined hybridization of ≥ 20 ‘double-Z’ target probe pairs. In addition to the exceptional specificity and sensitivity the RNAscope platform offers, this approach is remarkably rapid and facile, with results obtained in just 1 day (< 8 h) compared to up to 4 weeks with R-ISH [16, 23].

**Fig. 1:**
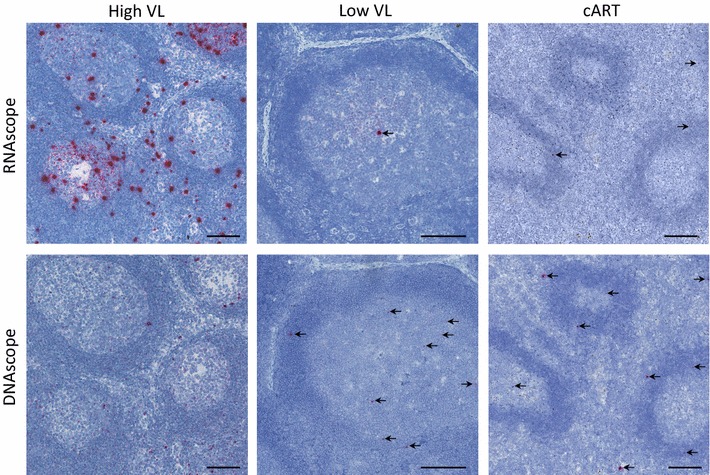
RNAscope and DNAscope ISH in tissue sections. Representative images of SIV RNAscope and DNAscope in lymph nodes from rhesus macaques during acute infection with a high viral load (VL) and robust levels of infected cells (left), chronic infection with low levels of infected cells (middle), and chronic infection under suppressive cART (right). Scale bars 200 μm

Due to its promise for detecting low abundant host RNA transcripts, we developed and optimized an RNAscope platform for the detection of HIV and SIV RNA in tissue sections [16]. We have designed RNAscope probes for HIV clades A, CRF_AE, B, C, and D, as well as probes for SIVmac239, SIVagm and SHIV-C1157; each contains ~ 80 ‘double-Z’ target probe pairs representing up to ~ 4.3 kb coverage of the HIV/SIV genome and targeting both 5’ and 3’ gene regions that bind to spliced and unspliced RNA transcripts [16]. Using this next-generation RNAscope platform, we demonstrated the specific detection of HIV and SIV vRNA^+^ cells and virions in tissue sections, with the visual resolution necessary to identify individual viral particles bound to the FDC network before and during cART, although the latter was present at greatly reduced levels (Fig. 1). The RNAscope approach had a trend towards greater sensitivity in detecting productively infected vRNA^+^ cells compared to both R-ISH and C-ISH but with remarkably lower background than the conventional ISH approaches [16]. Furthermore, RNAscope was similar to the gold standard R-ISH in detecting viral particles bound to cells, with both RNAscope and R-ISH being about 3 orders of magnitude more sensitive at detecting viral particles bound to cells than the C-ISH method; however, RNAscope demonstrated clearly superior visual discrimination of viral particles bound to cells, observed as discrete punctate signals compared to the diffuse silver grains surrounding cells using the R-ISH method [16]. Importantly, the RNAscope platform for the detection of HIV and SIV RNA can be fully automated using the BOND-RX (Leica Biosystem) or the Ventana Discovery Ultra/XT (Roche) autostainer systems. These two flexible and sophisticated automated platforms bring the benefits of increased consistency, reduced labor costs, and increased high-throughput capability.

In order for in situ based approaches to meaningfully augment our understanding of HIV/SIV reservoirs and viral persistence in vivo, like with vRNA detection, we must have an extremely specific and sensitive approach to detect vDNA in tissues. Previous in situ approaches aimed at vDNA detection have been hampered by numerous challenges that have limited their usefulness in reservoir studies (i.e. assay complexity, need for long exposure times, and challenges with reproducibility and background). Given our success in detecting particularly low abundant HIV/SIV vRNA in tissues, including viral particles that only contain two vRNA copies, we modified and optimized the RNAscope approach for the detection of HIV/SIV DNA by designing target probes specific for the sense/coding strand, an approach we now term ‘DNAscope’ (Fig. 1). We have demonstrated that DNAscope ISH can rapidly (in 2 days) and reproducibly resolve a single vDNA copy in the nucleus of infected cells in tissues [16]. Importantly, DNAscope ISH was consistently able to detect vDNA^+^ cells to nearly the level of sensitivity seen with quantitative PCR methods before and during cART [16], with the added advantage of retaining the contextual information of the infected cells in their native tissue microenvironment at the time of specimen collection.

**Fig. 2:**
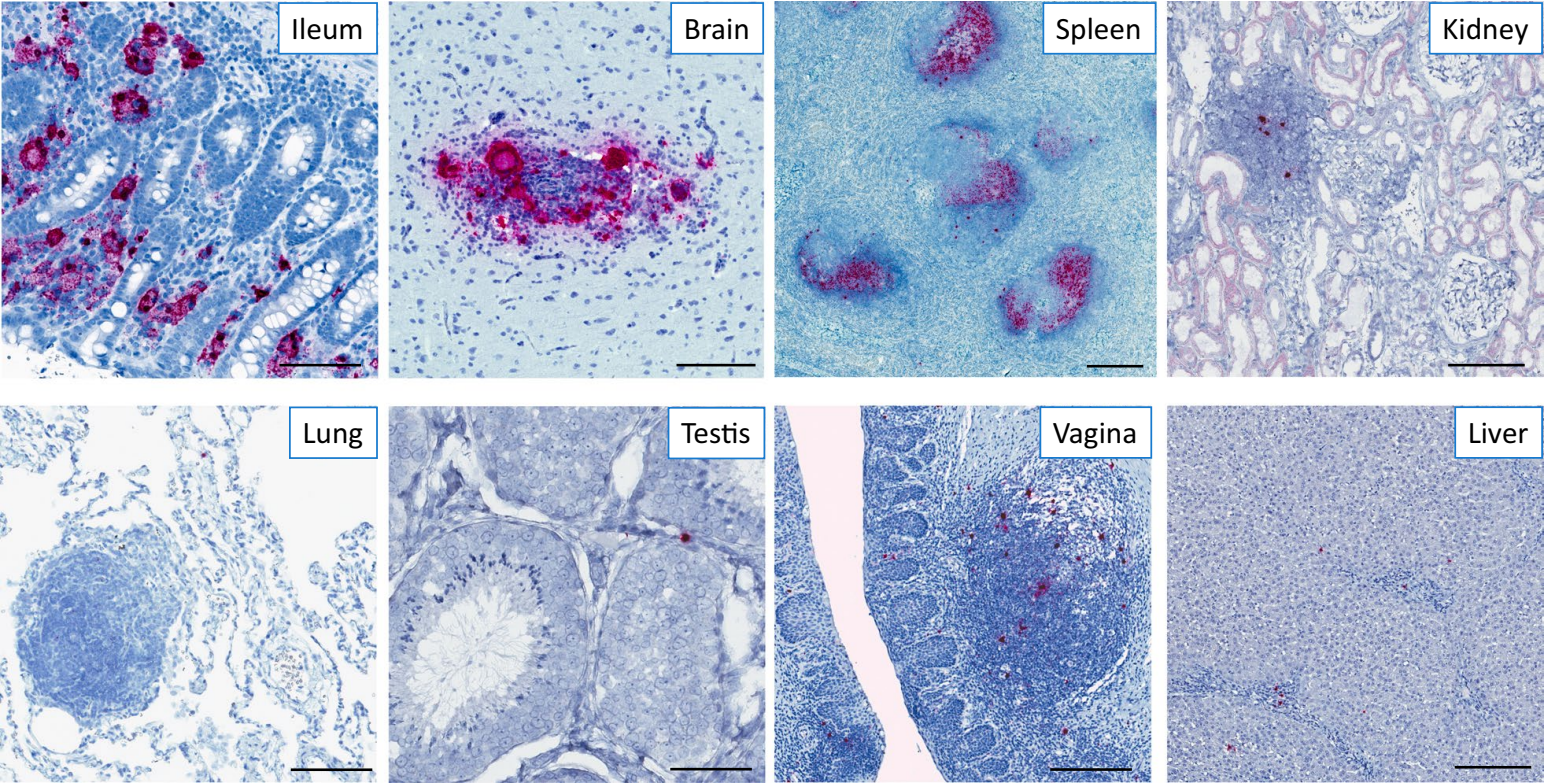
Flexibility and reproducibility of the RNAscope approach in different tissue types. Representative images of SIV RNAscope performed on chronically infected rhesus macaques in ileum, brain, spleen, kidney, lung, testis, vagina, and liver showing productively infected vRNA^+^ cells and viral particles on FDCs within the BCFs. Scale bars 200 μm

While CD4^+^ T cells are the primary target cell for HIV and SIV, with the vast majority of these cells residing within lymphoid tissue (i.e. lymph nodes, spleen, gut, lung, etc.), HIV and SIV can target several different cell types (i.e. macrophages, microglia, etc.) in numerous diverse tissue microenvironments [24–32]. In addition, infectious HIV can be trapped and retained as immune complexes for long periods of time on FDCs, which only reside within BCFs of lymphoid tissues [33–36]. The persistence of infected cells in a wide variety of tissues, as well as the large repository of HIV/SIV trapped on FDCs, emphasizes the importance of studying tissue reservoirs in the development and assessment of ‘cure’ strategies. Thus, the next-generation RNAscope and DNAscope ISH approaches must be capable of reproducibly detecting rare productively infected vRNA^+^ cells and vDNA^+^ reservoirs, and virions trapped on FDCs, in lymphoid and non-lymphoid tissues like the CNS, male and female genital tract, liver, and kidney (Fig. 2). The strength of the RNAscope and DNAscope platforms is the flexibility to use these approaches on all types of fixed (FFPE) or frozen (OCT) tissues with similarly high sensitivity and specificity, and low background. In contemplating in situ-based approaches for reservoir studies, careful consideration should be given to the experimental design; in particular, the amount of tissue needed to accurately quantify the reservoir must be critically evaluated. As the frequency of infected cells becomes rarer, the need to sample larger tissue areas correspondingly increases. As with many techniques (i.e. other methods or procedures requiring only a small sample volume such as ELISA, PCR, etc.), sampling multiple organs, as well as an increased number of tissue sections throughout each organ, decreases the likelihood of false negatives when using RNAscope and DNAscope approaches.

Recent work has illustrated the unique capabilities that modern ISH approaches provide in guiding research into HIV/SIV tissue reservoirs and strategies for eradication. The combination of visualizing vRNA in situ, coupled with the low limit of detection achievable with these next-generation ISH methods, has been crucial in demonstrating that BCFs serve as important ‘sanctuary sites’ for HIV/SIV, even in the absence of detectable virus in the blood [16, 37]. Elite controllers are individuals that naturally limit HIV/SIV infection; however, despite the robust systemic CD8^+^ T cell-mediated cytotoxic responses in elite controllers, vRNA can still be found in infected T follicular helper cells (T_FH_) and trapped on FDCs inside BCFs in lymphoid tissues, in contrast to the mixture of both paracortical T cell zone and BCF localization concomitantly observed in typical HIV/SIV progressors [37]. Since BCFs are not easily accessible to SIV-specific follicular cytotoxic CD8^+^ T cells (T_FC_) [38], this represents an opportunity for vRNA-producing T_FH_ cells to escape immune clearance.

Determining the precise location of vRNA-producing or vDNA-harboring cells, as well as the quantitative and proportional viral burden within key organ systems and tissue compartments in situ, via ISH, may help guide novel approaches for viral eradication. For instance, in a NHP model we recently demonstrated that the vast majority (> 98%) of vRNA^+^ and vDNA^+^ cells present in SIV-infected rhesus macaques (RMs) before and during cART-mediated viral suppression were primarily found within lymphoid tissues (i.e. lymph nodes, gut, lungs, and spleen) [24]. vRNA^+^ cells, while typically lower than in viremic animals, were identified in every major organ system in SIV-infected RMs during suppressive cART, with some tissues, like the CNS, not seeing measurable declines [24]. The estimated total number of vRNA^+^ cells within SIV-infected RMs during suppressive cART was on the order of 10^8^ cells in the body, suggesting a remarkably large pool of infected cells actively transcribing viral RNA during cART [24]. Moreover, an analysis of the ARV drug levels in mononuclear cells from lymphoid tissues harboring ISH-detectable vRNA^+^ cells, compared to PBMCs, suggests that ARV penetration within some tissues may be more challenging and less complete than previously thought [24]. Thus, the latent CD4^+^ T cell pool, in addition to large numbers of vRNA^+^ cells within select tissues during cART, perhaps in part due to lower ARV drug levels in combination with poor anti-viral control, indicate two important potential sources of virus that may rapidly reignite infection after treatment interruption. The large size and distribution of these viral reservoirs within an array of tissue compartments (albeit at variable sizes) underscores the challenges that will be faced in developing effective ‘HIV cure’ strategies that must penetrate and target multiple sources of infected cells that harbor replication competent virus.

### Multiplexing RNAscope and DNAscope

**Fig. 3:**
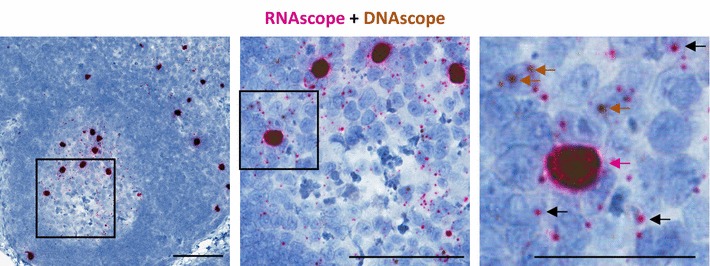
Multiplexing RNAscope and DNAscope on the same tissue section. Representative images with progressive magnification of multiplexed RNAscope and DNAscope on the same lymph node section from an SIV acutely infected rhesus macaque. In the final pane, the red arrow highlights a vRNA^+^/vDNA^+^ productively infected cell, the brown arrows highlight vRNA^−^/vDNA^+^ infected cells, and the black arrows highlight examples of the numerous SIV viral particles trapped on the FDC network. Scale bars 200 μm

The study of viral reservoirs, including latently infected resting CD4^+^ T cells, in their native tissue microenvironments requires the need to discriminate infected vDNA^+^ cells that are transcriptionally silent (vRNA^−^) from those infected cells that are actively transcribing viral RNA (vRNA^+^). Thus, we developed an approach that combines RNAscope and DNAscope to identify and localize vDNA^+^ cells expressing, or not expressing, vRNA in the same section of tissue [16] (Fig. 3). The multiplexing of RNA with DNA is not limited to only viral targets, as these approaches can be coupled with RNAscope-mediated detection of any cellular RNA for which target probes are available and have been validated. This approach would provide a sensitive approach for the detection of potentially important host genes expressed in target cells, like host anti-viral factors (i.e. APOBEC3G, Mx2, Trim5α, etc.) that have been demonstrated to play a role in attenuating HIV/SIV infection [39–41]. These combined approaches could provide important insights in understanding the mechanisms involved in viral reservoir establishment, maintenance, and reactivation in both NHP models and HIV-infected patient samples.

### Phenotyping infected cells in situ

While multi-color flow cytometric analysis of cells from peripheral blood and tissue homogenates has provided important phenotypic characterization of several HIV/SIV CD4^+^ T cell reservoir populations [9, 10], these approaches lose important contextual information of the infected cells in their native tissue microenvironments, including an understanding of the surrounding cell populations and immunological milieu within the tissue sites wherein the infected cells resided at the time of collection. In addition, RNA and protein expression levels may be altered or lost during the cell isolation procedure. Alternatively, in situ phenotypic studies of productively infected vRNA^+^ cells by RNAscope and vDNA^+^ cells by DNAscope can provide powerful additional insights into reservoir biology and mechanisms of viral persistence.

**Fig. 4:**
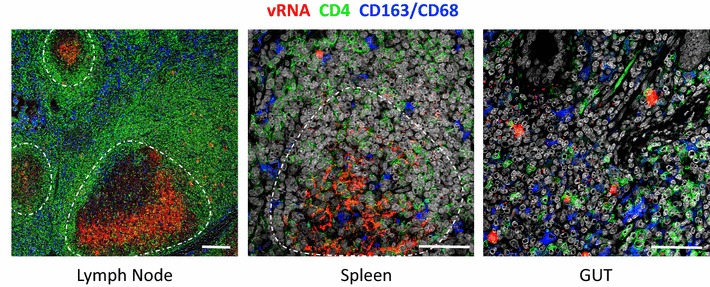
Combining RNAscope with immunofluorescent confocal analysis of specific cell protein markers. Representative images of lymph node, spleen, and gut combining RNAscope (red) with staining for CD4 (green) to identify T cells and a combination of CD163 and CD68 (blue) to identify myeloid/macrophage cells. Scale bars 200 μm

We have therefore developed additional approaches to combine RNAscope and/or DNAscope with immunofluorescent confocal analysis of specific cell protein markers to characterize productively infected vRNA^+^ and vDNA^+^ cells in situ within tissues (Fig. 4). This combination of precise localization of virus within tissues, coupled with the ability to discriminate cell populations based on protein expression, recently led to the identification of a hitherto unrecognized HIV/SIV reservoir population, CTLA4^+^PD-1^−^ memory CD4^+^ T cells [42]. In addition to the sensitivity that DNAscope provided that enabled detection of rare vDNA^+^ cells, staining for phenotypic markers in situ highlighted location differences between the previously-described PD-1^+^ T_FH_ population (B cell follicles), as compared to the newly-described CTLA4^+^PD-1^−^ memory CD4^+^ T cells (non-B cell follicle), which had a regulatory T cell-like phenotype. Since tissue penetration of ARV and/or ‘HIV cure’ therapies may occur in a highly site-specific manner [24], as previously mentioned, a better understanding of the predominate location and phenotype of viral reservoirs within tissue microenvironments may help guide the development of successful ‘HIV cure’ strategies.

## Future in situ tools

### Combining RNAscope/DNAscope with histo-cytometry

Flow cytometry provides a powerful tool for the quantitative phenotypic (and potential effector functional) analysis at the single cell level at very high throughput, allowing for the detailed interrogation of multiple complex cellular populations. However, as mentioned above, this comes at the cost of losing important contextual information about the cells in their native tissue microenvironments. On the other hand, while immunofluorescent in situ imaging for the phenotypic characterization of cells in tissue sections retains cellular contextual information, this approach has historically been limited by the number of markers that could be discriminated by confocal microscopes. The capacity to perform detailed quantitative immunophenotypic analysis in situ was significantly enhanced with the advent of an approach, termed ‘histo-cytometry’, that combines the quantitative expression analyses of multiple phenotypic markers typically restricted to flow cytometric analysis of disassociated single cell suspensions, with the valuable anatomical location information provided by confocal microscopy [43–45]. Since this new analytical approach achieves quantitatively similar results to flow cytometry, while retaining detailed cellular positional information in situ, merging the highly sensitive RNAscope and DNAscope approaches with histo-cytometry holds great promise in obtaining new detailed immunophenotypic information of viral reservoirs in vivo.

### Detection of spliced RNA using BaseScope ISH

Like most current approaches utilized to quantify HIV/SIV reservoirs, these next-generation ISH approaches do not discriminate between replication competent genomes capable of producing infectious virus leading to de novo infections and defective genomes that result in the production of non-infectious virions. The inability to determine the capacity of an infected cell that is either actively transcribing viral RNA (vDNA^+^/vRNA^+^) or transcriptionally silent (vDNA^+^/vRNA^−^) to produce infectious virus in situ remains a major limitation in the characterization and quantification of HIV/SIV reservoirs in tissues. A new approach developed by Advanced Cell Diagnostics (ACD), termed BaseScope™ [46], extends the established RNAscope technology and may hold some promise in getting closer to the goal of detecting infected cells capable of producing replication competent virus. The BaseScope™ approach enables applications such as the detection of exon splice junctions in FFPE tissue with morphological context and the ability to visualize gene expression of short RNA target sequences (as short as 50 bp) with as few as one ‘double-Z’ target probe pair. This new, more sensitive approach could be used to target regions in the 3′ HIV/SIV genome that have been demonstrated to be deleted or hypermutated in > 70% of full-genome sequences analyzed in HIV-infected individuals on cART (i.e. the 3′ tat-rev splice junction) [47–49], identifying with greater confidence vRNA from infected cells that may have the ability to produce infectious virus.

This additional next-generation approach could be used to better understand and dissect the HIV/SIV life cycle, including temporal regulation and balance between the at least 20 different splicing events necessary to generate mRNAs encoding accessory proteins, viral polyproteins, and genomic vRNA in situ. In addition, because of the variability in the HIV/SIV consensus splice acceptor sequence, this new approach could allow for an increased understanding of alternative splicing events within infected cells in tissues in vivo for the first time.

## Summary

**Fig. 5:**
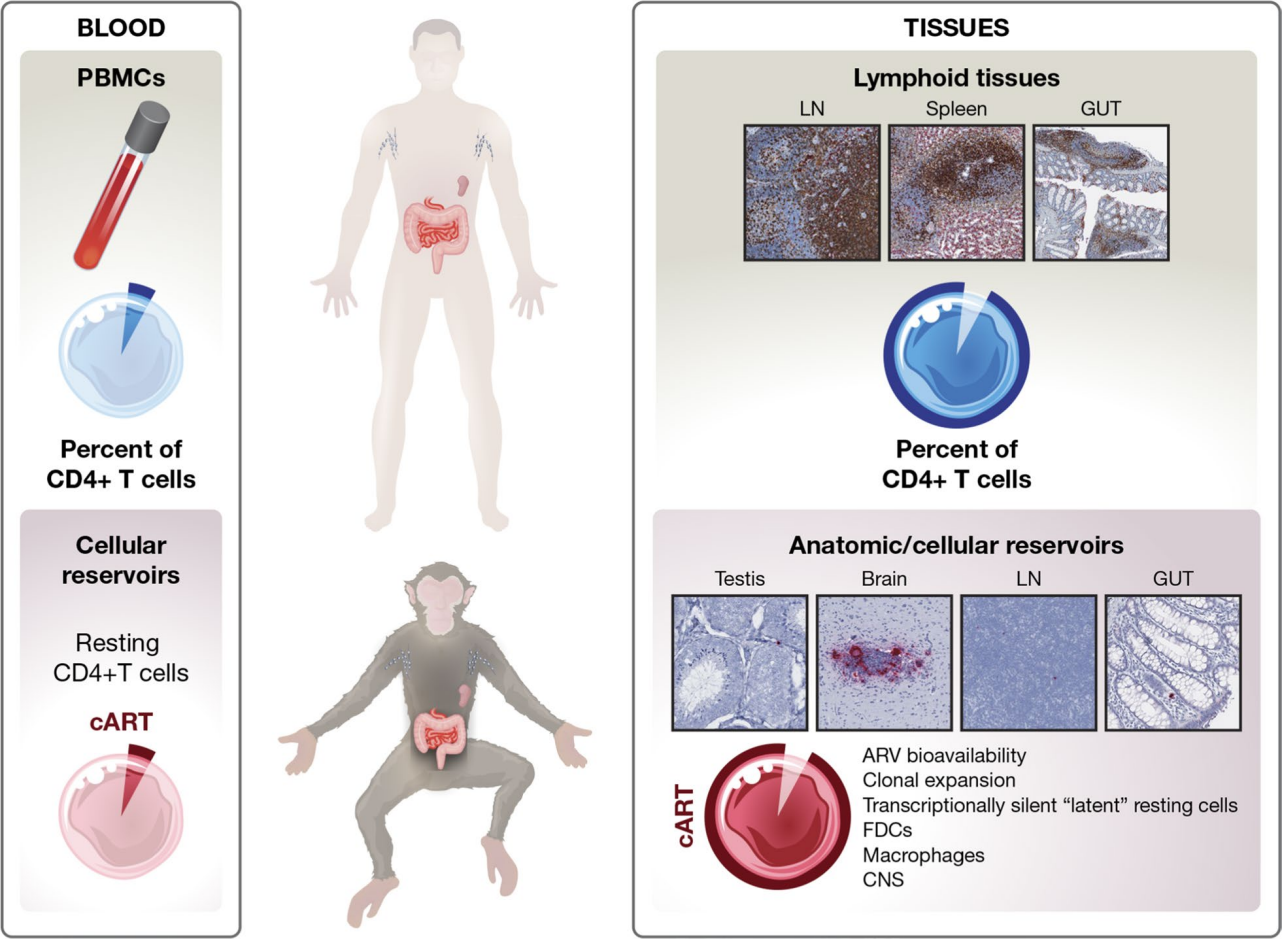
Cartoon schematic summarizing the importance of lymphoid tissues in harboring the vast majority of the infected cellular reservoir before and during effective cART treatment. A very small fraction of potential target cells is found in the peripheral blood compared to lymphoid tissues (i.e. lymph nodes, spleen, gut). Both before and during cART, the localization of infected cells (and/or trapped virions on the FDC network) within lymphoid tissues account for a disproportionately vast majority of infected cells in the body. Modern ISH approaches have been important in understanding unique tissue compartments where infected cells reside and persist during cART, like BCFs of lymph nodes, gut, reproductive organs (like testis), and CNS. Mechanisms contributing to viral persistence within tissue compartments are likely multifactorial, and may include: variable tissue ARV drug penetration, continued virus production by infected cells, and/or homeostatic and/or antigenic clonal expansion of infected cells. Collectively, ISH approaches have made important contributions to our understanding of viral persistence in tissue compartments and to reservoir biology, and illustrate the relevant need to focus efforts on developing novel methods to explore tissue reservoirs for the assessment of ‘HIV cure’ strategies

The mechanisms responsible for HIV/SIV persistence in T cells are thought to be multifactorial, and include: (1) HIV transcriptionally quiescent (latent) resting memory CD4^+^ T cells receiving survival signals as they circulate through the periphery and immune organs, (2) homeostatic and/or antigenic proliferation of latent or active memory CD4^+^ T cell reservoirs, and (3) HIV integration site-driven cellular proliferation and expansion [50]. By virtue of their function, and containing the vast majority of the CD4^+^ T cells in the body, lymphoid tissues play a central role in the establishment and maintenance of the HIV/SIV reservoir pool (Fig. 5). A more detailed and comprehensive understanding of the anatomic and cellular reservoirs within tissue microenvironments will likely be needed to realize the full effectiveness of ‘HIV cure’ strategies. To date, the use of high-throughput, next-generation ISH has produced exciting contextual information about the location and cellular components of the HIV/SIV reservoirs. However, we are only just beginning to explore the full potential this current ISH technology offers. The strength of modern ISH approaches (i.e. a high degree of sensitivity and specificity) combined with in situ phenotyping will allow for a deeper understanding of rare viral reservoirs that may open the door to potential novel therapeutic paths.
